# Initial characterization of print awareness in unhoused children

**DOI:** 10.3389/fpsyg.2023.1274777

**Published:** 2024-02-12

**Authors:** Anne Hoffmann, Lauren Little, Kristen Vincent, Karen Lui, Laura Pabalan

**Affiliations:** ^1^Department of Communication Disorders and Sciences, Rush University Medical Center, Chicago, IL, United States; ^2^Department of Pediatrics, Rush University Medical Center, Chicago, IL, United States; ^3^Department of Occupational Therapy, Rush University Medical Center, Chicago, IL, United States; ^4^Department of Psychiatry and Behavioral Sciences, Rush University Medical Center, Chicago, IL, United States

**Keywords:** literacy, unhoused children, homelessness, print awareness, school readiness

## Abstract

This study provides an initial understanding of print awareness, a foundational literacy skill, in a group of 12 unhoused children at two shelters in a large urban setting. Children ranged in age from 4;1 to 8;0, representing grades associated with learning to read (i.e., pre-kindergarten to second grade). Findings indicate that the majority of children in this sample were significantly delayed in their acquisition of print awareness skills. Caregivers were surveyed regarding their beliefs about supporting literacy development and what would be beneficial for helping them in this area. Responses indicated that almost all caregivers believed that some literacy development should occur outside of the school setting and that it would be helpful if they (the caregivers) were taught both what skills to teach and how to teach them.

## 1 Introduction

Children experiencing homelessness (CEH) during early development often demonstrate decreased long-term academic outcomes, partially due to poor literacy outcomes. There is strong evidence that children who demonstrate delays in reading acquisition during their first three years of school typically do not catch up to their peers later in school (Knapp and Winsor, 1998; Willard and Kulinna, 2012). Disparities in reading ability are often apparent by third grade, when CEH have decreased school-readiness skills compared to the general population and score lower on academic achievement tests (Institute for Children, Poverty, and Homelessness, 2019, 2020; Manfra, 2019). Over 2.5 million children in the United States experience homelessness in a given year; approximately half are under the age of 6 years. In the 2017–2018 school year, approximately 1.28 million children enrolled in public schools were experiencing homelessness (Department of Housing and Urban Development, 2020). According to recent figures, 12,913 children under the age of 14 years experienced homelessness in Chicago alone in 2019 (Mendieta and Carlson, 2021). There are clear racial inequities in factors leading to homelessness. As of these children, the proportion identifying as Black was 74% (Medieta & Carson, 2021). This is radically different than the demographics of Chicago as a whole, where 29.2% of residents identify as Black (United States Census Bureau, 2022). Further, of those mothers under the age of 18, 93.4% identify as Black. These rates are indicative of the structural racism and socioeconomic disadvantage that puts Black women and children at a higher risk for homelessness, contributing to educational inequity (Jones, 2016).

### 1.1 Literature overview

There is growing research into the risk factors that systemic inequities create for under-resourced populations. This is particularly salient for CEH, as they exist in the intersection of multiple marginalized groups (e.g., low-income households, more likely to be from minority racial/ethnic groups) (e.g., Chiu and DiMarco, 2010). The gaps in school readiness demonstrated by CEH have been recognized for decades (Bassuk et al., 1986; Bassuk and Rubin, 1987; Lewis and Meyers, 1989; Rafferty and Shinn, 1991; Rescorla et al., 1991; Buckner, 2008). Research demonstrates that the risks associated with homelessness or increased residential mobility are apparent even before children enter the school system, and they continue throughout elementary school (Obradović et al., 2009). These children are also less likely to attend early childhood centers or be identified by the Early Intervention system, compounding their risk for poor developmental and academic achievement (National Research Council and Institute of Medicine, 2010).

Decreased literacy ability has far-reaching ramifications. Students with low reading ability are more likely to drop out before graduating high school and only 12% of children identified as being delayed in reading when they enter kindergarten will attend college (Children’s Reading Foundation). In adulthood, low literacy is associated with lower income (Ritchie and Bates, 2013) and increased rates of incarceration (Greenberg et al., 2007). Adults with low literacy attainment have been found to have lower salaries and decreased health literacy, resulting in higher risk for poor health outcomes (Berkman et al., 2011).

Previous research has clearly demonstrated that factors such as poverty and increased housing mobility place children at risk of not mastering the literacy skills necessary for academic success (Chaney, 1994; Lonigan et al., 1999; Smith et al., 2021). One reading domain that is crucial for literacy attainment is print knowledge, or an individual’s knowledge of the forms and functions of written language (Whitehurst and Lonigan, 1998; Storch and Whitehurst, 2002). This area of literacy is one that includes understanding how print is organized, the different functions that print may serve, the names and distinguishing features of alphabet knowledge, and how meaning and orthography is expressed through writing. These concepts are often learned prior to formal education and have been shown as more influenced by the environment than other areas of early literacy, such as phonological awareness (Petrill et al., 2005; Lemelin et al., 2007). Other aspects of the environment that have been shown as especially important for print knowledge development include caregiver involvement in their children’s school work and their children’s enjoyment in reading (Petrill et al., 2005), caregiver valuation of home literacy activities (Bassuk et al., 1986; Skibbe et al., 2008), the frequency caregivers read to their children (Bus et al., 1995; Sénéchal et al., 1998), and the quality of those shared readings (Scarborough and Dobrich, 1994). As many of these environmental factors may be challenging in the setting of a homeless shelters (i.e., decreased access to books, limited space for shared reading, etc.), unhoused children are at a disadvantage for mastering print knowledge. However, this same predisposition to be influenced by the environment means that print knowledge is also responsive to interventions that increase exposure to key elements of print (Justice et al., 2008), emphasizing the importance of early identification.

Given this confluence of risk factors for poor long-term outcomes, research is needed to examine literacy development among CEH as well as information about what families believe could support children’s growth in literacy. To date, no published studies have examined print knowledge ability in CEH. While studies with similar populations (e.g., children experiencing poverty) have demonstrated delays in print knowledge, the current study is the first to examine print awareness specifically in a group of children and caregivers experiencing homelessness.

## 2 Method

### 2.1 Research design and study overview

This study focused on establishing an initial understanding of the print knowledge skills of unhoused children. Participants were recruited from two shelters in a large urban setting. One shelter provides housing for women and children, intact families (both parents and children), and single men and women. The other shelter focuses on supporting women and children who have been victims of domestic abuse. Each shelter provides residents with case management, mental health counseling, parenting classes, and after-school programs for children.

### 2.2 Participants

Eligible participants were children between the ages of 4 years and 8 years 0 months. This age range was chosen to allow understanding of school-readiness literacy skills for children either about to enter formal schooling or in the early years when learning to read is still a primary goal. A total of 12 children enrolled in the study between the two shelters. Mean age of the participants was 5;8 with standard deviation of 1.6 and a range of 4;1 to 8;0. Caregivers identified three of the children as Hispanic and nine as Black. Each of the children was identified as using English as their primary language. Those who identified as Hispanic were reported to have some exposure to Spanish. All children were enrolled in English-only classrooms. No child was reported as either currently or previously receiving support services (e.g., speech therapy, resource). Those children considered school-age (i.e., 5 years or older by the start of school) were enrolled in local public schools. No data were available regarding attendance or previous school enrollments. We received approval for the current study from our university’s institutional review board and collected data between July 2022–March 2023.

### 2.3 Measures

To measure children’s print knowledge skills, three standardized criterion-referenced tools were used. All measures were administered in English. Each measure was individually administered to children in a private setting within their respective shelters by either the first or second author or a trained and supervised graduate student. All three measures have served as outcome measures for children in prior intervention research and have adequate psychometric properties (e.g., Justice and Kaderavek, 2003; Justice et al., 2009). We used the Upper-Case Alphabet Knowledge, Name-Writing Ability, and Print and Word Awareness subtests of the Phonological Awareness Literacy Screening: PreK (PALS-PK; Invernizzi et al., 2004). The alphabet measure is administered by asking children to produce the name of all 26 letters presented in random order on a large sheet of paper. Children receive one point for each correct letter (range = 0–26 points). The name writing measure asks children to produce a self-portrait and then to sign it. Children’s name-writing representations are scored on a 7-point scale based on a developmental continuum of early writing development; with a score of 1 for writing that is indistinct from pictures, and a score of 7 for writing that is legible and orthographically complete. The print and word awareness subtest asks children to point to various text components in a common nursery rhyme that is presented in a book format (e.g., “where is the title?”). Additionally, to assess caregiver’s comfort and knowledge with supporting literacy, we created a 10 question survey which was administered to each family. These questions assessed the following areas: how the family currently accessed books, the caregiver’s view of how literacy was taught (e.g., only in the school, within the household and the school), how comfortable the caregiver felt in supporting literacy development, and what supports the caregiver felt would be beneficial for encouraging literacy. These questions were chosen based on prior research indicating that children’s development of print knowledge is heavily influenced by parental beliefs concerning the benefits of home literacy activities (Bassuk et al., 1986; Skibbe et al., 2008); how often caregivers can access reading material (Bus et al., 1995; Sénéchal et al., 1998) and the quality of book-sharing experiences between a parent and child (Scarborough and Dobrich, 1994). The full survey can be seen in Supplementary Appendix S1.

## 3 Results

### 3.1 Print knowledge skills

**Figure 1:**
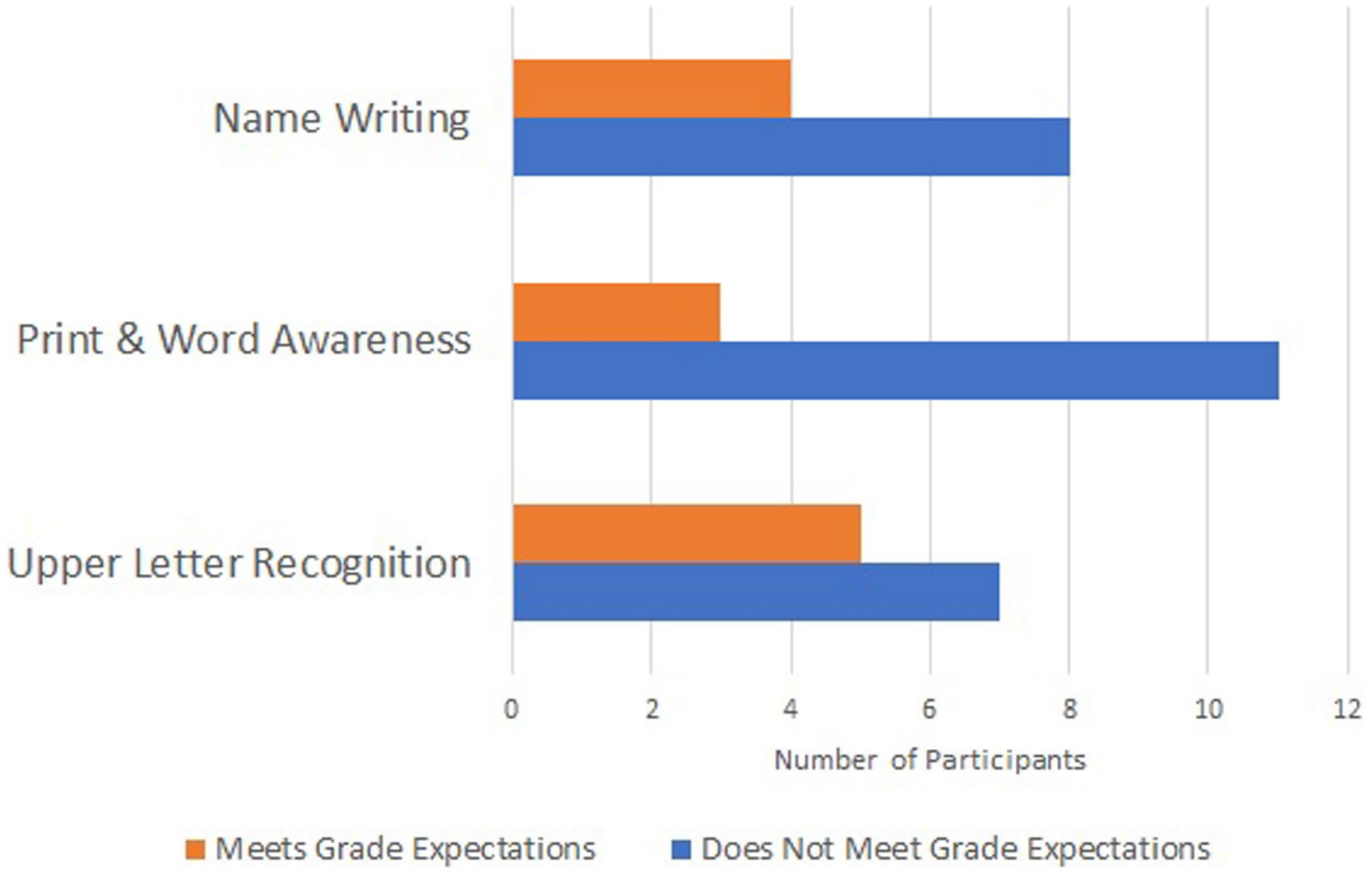
Literacy skills among participants.

For analysis, we divided the 12 participants into groups based on the grade in which they would be placed. Six students were in their 4-year-old/pre-kindergarten year, one was in kindergarten, two were in first grade, and three were in second grade. We then took the score that the PALS-PK considers to be at-risk of reading delay at the mid/end of pre-kindergarten and applied it to those students. For the later grades, we considered grade expectations as follows based on interpretation of the PALS-PK recommendations for students exiting their pre-kindergarten year and other recommendations for print skills (Lomax and McGee, 1987; Worden and Boettcher, 1990): Upper-Letter Recognition, students in pre-kindergarten should recognize at least 1 upper-case letters; students in kindergarten should recognize at least 21 letters, those in first grade and later should recognize all upper-case letters. Print-Word Awareness: students in pre-kindergarten should receive a scores of at least 7, students in kindergarten should receive a score of at least 9, those in later grades should receive a score of 10. Name Writing, students in pre-kindergarten should receive a score of at least 5, students in kindergarten and later should receive a score of 7. The performance of all students as compared to grade expectations is shown in Figure 1.

As shown below, based on these expectations, in Upper-Letter Recognition, 42% (5/12) students reached grade expectations. In Print and Word Awareness, 25% (3/12) students met grade expectations. In Name Writing, 33% (4/12) students reached grade expectations. In other words, the majority of children in this sample (58–75% depending on the specific skills assessed) were delayed in their acquisition of early reading milestones. A Spearman-Rho correlation was calculated to determine if there was a relationship between age and the likelihood of meeting grade expectations. This revealed no significant correlation between age and achievement of grade-level literacy skills (*ρ* = 0.389, *p* = 0.211). The three children with multiple language exposure had similar performance to their monolingual peers. There was a higher percentage of success in those more widely recognized academic skills such as letter recognition (42%) as compared to less commercially targeted skills such as separating words and text organization (25%).

### 3.2 Caregiver view of needed literacy supports

**Figure 2:**
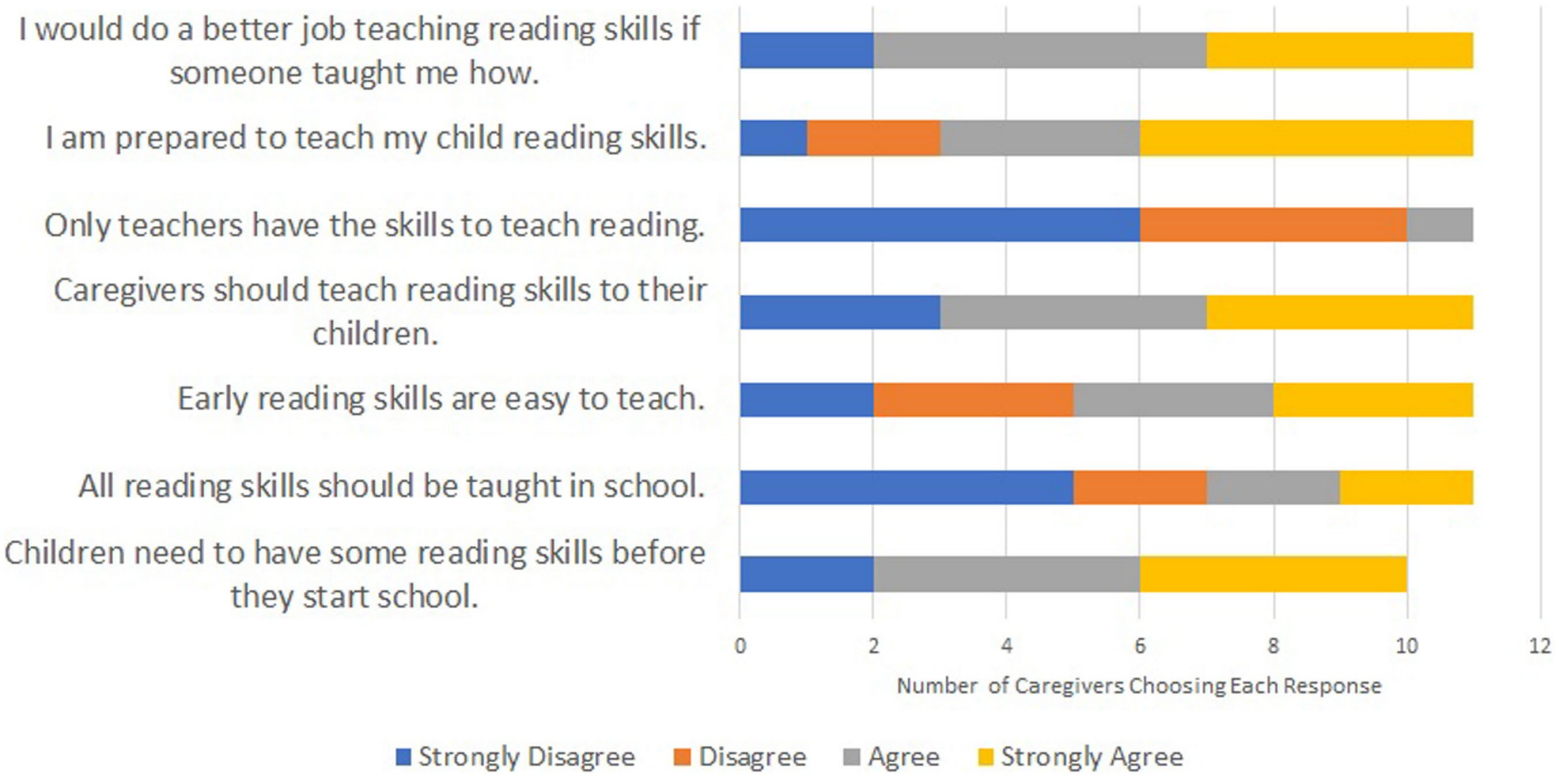
Caregivers’ perceptions of reading supports.

Ten caregivers completed the literacy survey, each was the mother of a child participating in the study. Results are shown in Figure 2. The responses indicated that caregivers mostly agreed that children should have some reading skills before starting school, that caregivers should teach some of these skills, and that most caregivers believed that they would be more effective in supporting literacy development if someone told them what skills their children should be learning and how to support those. Caregivers disagreed with the statement that reading skills should only be taught in schools or by teachers.

When asked what supports would be helpful, caregivers most highly reported that having more access to books would most support children’s literacy development. Surprisingly, having a quiet location to work on reading and/or having more time with their child were not frequently cited as beneficial changes.

## 4 Discussion

This study provides an initial understanding of print knowledge ability in a group of unhoused children, and relays novel findings related to print knowledge ability early in development among CEH. This is also a snapshot of early academics in CEH who experienced the secondary effects of the COVID-19 pandemic during critical periods of development. Research has established a substantive gap in the academic readiness of CEH (Herbers et al., 2012; Fantuzzo et al., 2013; Manfra, 2019), which is magnified for children experiencing homelessness early in life (Barnes et al., 2017; Kanak et al., 2018). However, previous studies have examined academic readiness holistically and measured it among those later in development. While estimates vary, some studies suggest that 47–63% of CEH show significant delays in language and communication skills (Haskett et al., 2016; Coughlin et al., 2020) and up to 78% show academic delay, which is largely contingent on language skills (Bassuk et al., 1986; Bassuk and Rubin, 1987; Lewis and Meyers, 1989; Rafferty and Shinn, 1991; Rescorla et al., 1991; Zima et al., 1994; Buckner, 2008).

The current findings align with previous findings, suggesting that, across subscales associated with early literacy ability, only 25–42% of these children demonstrated grade level expectations depending on the task. Thus, the majority of children in our current sample do not demonstrate grade level skills in basic foundations of reading. The cascading effects of foundational reading deficits early in life often contribute to decreased academic outcomes throughout development. This means that providing literacy interventions early in life for those most at risk, including unhoused children, may be highly effective and cost effective in ameliorating academic delays.

The effects of homelessness are particularly acute during early, critical periods of child development; children who demonstrate delays in reading acquisition during their early childhood typically did not catch up to peers in middle school (Knapp and Winsor, 1998; Willard and Kulinna, 2012). Those students who do not meet early reading milestones are at a heightened risk for poor outcomes, including dropping out prior to receiving a high school diploma, increased risk of incarceration, and decreased health literacy leading to poor health outcomes. As children experiencing homelessness are already at risk because of other risk factors (e.g., low socio-economic status, minoritized racial/ethnic status, housing instability), identification of potential reading delay and then prompt and aggressive intervention is clearly indicated.

Literacy interventions have been shown to increase caregiver involvement in literacy development and increased reading achievement among children experiencing poverty (Justice and Kaderavek, 2003; Justice et al., 2009; Willard and Kulinna, 2012; Di Santo et al., 2016). Sinatra (2007) provided 240 students residing in transitional facilities with a 10 day literacy intervention program over a period of 3 weeks, and found significant gains in writing skills and children’s self-perceived abilities as readers. Another intervention that asked parents in a homeless shelter about their feelings about literacy both before and after a conversation about the importance of reading found that the conversation made mothers more likely to express an intention to read to their children (Petruccelli et al., 2016). Other interventions with youth who have many of the same risk factors as CEH (e.g., limited resources, low parental education) have demonstrated that commonly recommended literacy enhancing methods can be effective (Justice et al., 2008). Further, as part of the intervention development process, caregivers were surveyed regarding their support needs. The biggest areas of need identified by these families were knowing what skills to teach and how to teach them. This indicates that an intervention that includes a caregiver component could increase caregiver self-efficacy in supporting early reading development. Future research should expand our understanding to what caregivers view as barriers to accessing support for early literacy development. Given the multiple stressors experienced by families with housing insecurity, it is likely that a more varied approach to supporting them may be necessary (e.g., training community members to assist with intervention, providing childcare for siblings).

## Data availability statement

The raw data supporting the conclusions of this article will be made available by the authors, without undue reservation.

## Ethics statement

The studies involving humans were approved by Rush University Institutional Review Board. The studies were conducted in accordance with the local legislation and institutional requirements. Written informed consent for participation in this study was provided by the participants’ legal guardians/next of kin.

## Author contributions

AH: Conceptualization, Formal analysis, Funding acquisition, Investigation, Methodology, Project administration, Writing – original draft. LL: Conceptualization, Formal analysis, Methodology, Project administration, Writing – original draft. KV: Investigation, Writing – review & editing. KL: Writing – review & editing, Conceptualization, Funding acquisition. LP: Writing – review & editing, Conceptualization, Funding acquisition.
