# Supplementary material for: Initial characterization of print awareness in unhoused children
Source: Front Psychol. 2024 Feb 12;14:1274777. doi: 10.3389/fpsyg.2023.1274777 (PMC10897729; doi:10.3389/fpsyg.2023.1274777)
Supplement: Supplementary file 1 [file Data_Sheet_1.PDF]

Caregiver Literacy Survey

1. What supports would help me teach my child reading skills (circle all that apply)
   a. More available books
   b. Knowing what skills to teach
   c. Learning how to teach different skills
   d. More time to spend one on one with my child
   e. A quiet location to work on reading
   f. I have all the supports I need
   g. There are no supports that can help
   h. Other (please list)

2. How does your family access books?
   a. We have books in our room
   b. We borrow books at the library
   c. We get books from school
   d. We get books from other sources (e.g., the shelter, friends)
   e. We do not have access to books
   f. Other (please list)

3. Children need to have some reading skills before they start school
   a. I strongly disagree
   b. I disagree
   c. I agree
   d. I strongly agree

4. All reading skills should be taught at school
   a. I strongly disagree
   b. I disagree
   c. I agree
   d. I strongly agree

5. Early reading skills are easy to teach
   a. I strongly disagree
   b. I disagree
   c. I agree
   d. I strongly agree

6. Caregivers should teach reading skills to their children
   a. I strongly disagree
   b. I disagree
   c I agree
   d. I strongly agree

7. Only teachers have the skills to teach reading
   e. I strongly disagree
   f. I disagree

g. I agree
h. I strongly agree

8. I am prepared to teach my child reading skills
   a. I strongly disagree
   b. I disagree
   c. I agree
   d. I strongly agree

9. I would do a better job teaching reading skills if someone taught me how
   a. I strongly disagree
   b. I disagree
   c. I agree
   d. I strongly agree

10. I would do a better job teaching reading skills if I had more access to books
   a. I strongly disagree
   b. I disagree
   c. I agree
   d. I strongly agree
